# Maternal PTSD and Depression as Predictors of Child Internalizing and Externalizing Symptoms: The Mediating Roles of Parenting Stress and Maternal Mentalization

**DOI:** 10.3390/healthcare14080984

**Published:** 2026-04-09

**Authors:** Rossella Procaccia, Giulia Segre, Cristina Liviana Caldiroli

**Affiliations:** Department of Theoretical and Applied Sciences (DiSTA), eCampus University, 22060 Novedrate, Italy; giulia.segre@gmail.com (G.S.); cristinaliviana.caldiroli@uniecampus.it (C.L.C.)

**Keywords:** maternal PTSD, maternal depression, internalizing and externalizing problems, mentalization, parenting stress, intimate partner violence, intergenerational trauma

## Abstract

**Background:** Exposure to intimate partner violence (IPV) represents a major risk factor for both maternal psychological well-being and child development. Maternal psychopathology—particularly depression and post-traumatic stress disorder (PTSD)—has been shown to impair parenting functioning and increase children’s vulnerability to emotional and behavioral difficulties. **Objectives:** This study examined the associations between maternal depression and PTSD symptoms and children’s internalizing and externalizing problems, and explored whether parenting stress and maternal mentalization capacities mediate these relationships. **Methods:** The sample included 42 mothers (mean age = 43.38, SD = 10.56) and their preschool- and school-aged children (n = 42; mean age = 8.30, SD = 2.53) exposed to IPV. Mothers completed self-report measures assessing depressive and PTSD symptoms, parenting stress, and mentalization (uncertainty and certainty about mental states). Children’s internalizing and externalizing problems were assessed through maternal report. Mediation analyses with bootstrapping procedures were conducted to examine indirect effects. **Results:** Maternal depressive symptoms emerged as the strongest predictor of children’s internalizing problems. Parenting stress was associated with stronger relationships between maternal symptoms and children’s internalizing problems, while polarized mentalization—particularly uncertainty and, to a lesser extent, excessive certainty about mental states—partially mediated the relationship. Maternal PTSD symptoms predicted both internalizing and externalizing problems. Parenting stress fully mediated the association between PTSD symptoms and children’s externalizing behaviors, whereas excessive certainty and uncertainty about mental states showed partial mediation effects. **Conclusions:** These findings suggest that maternal psychopathology may influence child adjustment both directly and indirectly through increased parenting stress and dysregulated mentalization. The results highlight the importance of trauma-informed, dyadic interventions targeting maternal mental health, parenting stress, and reflective functioning to prevent the intergenerational transmission of trauma and support resilience in families exposed to IPV.

## 1. Introduction

Intimate Partner Violence (IPV) refers to “any behavior within an intimate relationship that causes physical, sexual, or psychological harm to one of the partners. It includes physical assault, sexual coercion, emotional abuse, and controlling behaviors” [1]. IPV typically manifests as a recurring pattern of actions—most often perpetrated by men against women—aimed at establishing dominance and maintaining control [2,3]. It may be enacted by current or former partners, regardless of cohabitation status, and encompasses not only physical violence and threats, but also psychological tactics designed to instill fear, confusion, and self-doubt. These behaviors frequently involve financial control, social isolation, coerced substance use, forced illegal activities, and threats or violence directed at loved ones [4].

IPV is a pervasive global public health concern that transcends socioeconomic and cultural boundaries. According to the World Health Organization [5], nearly one in three women worldwide has experienced physical and/or sexual violence by an intimate partner during her lifetime [4]. Beyond its immediate physical consequences, IPV is increasingly conceptualized as a form of chronic relational trauma characterized by coercive control, fear, and emotional manipulation [2]. Its cumulative nature is associated with long-term psychological, relational, and developmental consequences that may extend across generations [6,7,8].

### 1.1. Maternal Psychological Sequelae and Intergenerational Consequences

Extensive research has documented the broad range of physical and psychological outcomes associated with IPV. Survivors face increased risks of injury, chronic pain, migraines, gastrointestinal disorders, gynecological complications, and sexually transmitted infections (i.e., [9,10]). Mental health consequences include substance misuse, insomnia, anxiety, mood disorders, and suicidal ideation (i.e., [10,11]). Post-traumatic stress disorder (PTSD), depression, and anxiety disorders are particularly prevalent among women exposed to IPV [12].

The chronic and interpersonal nature of IPV intensifies trauma-related symptoms such as hyperarousal, intrusive memories, and persistent fear and helplessness [13]. PTSD and depression frequently co-occur, reflecting shared mechanisms including emotional dysregulation, negative self-concept, and diminished sense of agency [14]. Feelings of guilt and shame may further contribute to avoidance, social withdrawal, and emotional numbing. Prolonged exposure to coercive control can also result in learned helplessness, characterized by passivity and resignation even after the abuse has ended [15,16,17].

At the neurobiological level, IPV-related trauma has been associated with dysregulation of the hypothalamic–pituitary–adrenal (HPA) axis and alterations in neural circuits involving the amygdala and prefrontal cortex, impairing stress responsivity and emotion regulation [18,19,20]. These changes have direct implications for maternal functioning. Maternal PTSD and depression are consistently linked to less responsive, more intrusive, or inconsistent parenting behaviors, which in turn increase children’s vulnerability to socioemotional and behavioral difficulties [21,22].

IPV is more prevalent in households with children, and its impact extends significantly to offspring. Children may be exposed directly through witnessing violence or indirectly through maternal distress and disrupted caregiving. Studies show that IPV-exposed children display elevated rates of internalizing symptoms (e.g., anxiety, depression, withdrawal, somatic complaints) and externalizing behaviors (e.g., aggression, defiance, conduct problems) [7,23,24,25,26].

Maternal PTSD and depression have been shown to mediate the association between IPV exposure and child psychopathology, highlighting maternal functioning as a central mechanism in the intergenerational transmission of trauma [27,28,29]. Moreover, children of IPV survivors often exhibit biological markers of chronic stress, including heightened cortisol levels and altered autonomic reactivity, consistent with the conceptualization of IPV as a form of toxic stress that disrupts neurodevelopment and emotional self-regulation. Collectively, these findings indicate that IPV affects mothers and children through interconnected emotional, relational, and physiological pathways [30,31,32].

### 1.2. Theoretical Models of Parenting in IPV-Exposed Mothers

The intergenerational transmission of trauma framework provides a central lens for understanding how IPV-related trauma influences parenting and child development. This perspective posits that traumatic experiences in one generation can shape the psychological and emotional functioning of subsequent generations, even in the absence of direct trauma exposure [33].

In violent relationships, mothers—often primary attachment figures—may experience significant psychological compromise, contributing to unstable and unpredictable family environments. Children may internalize maternal distress, assume inappropriate emotional responsibilities, or develop maladaptive relational patterns that hinder socioemotional development [34].

Research examining parenting among IPV survivors has yielded mixed findings. While some studies report reduced warmth, sensitivity, and emotional availability alongside increased harshness or neglect, others document heightened protectiveness or responsiveness [35,36,37,38].

Several theoretical models have been proposed to account for these variations in parenting among IPV-exposed mothers. The spillover model suggests that emotional distress originating within the partner relationship transfers into the parent–child relationship, resulting in irritable, harsh, or emotionally withdrawn caregiving [7,39,40]. Similarly, the traumatic reactivation model emphasizes the impact of unresolved trauma, proposing that children’s everyday behaviors—such as crying or defiance—may activate trauma-related memories in the mother, leading to hyperarousal, avoidance, or inconsistent caregiving responses [41,42].

From an attachment perspective, these processes can be understood in terms of disruptions in the caregiving system: when caregivers are themselves traumatized or emotionally dysregulated, their capacity to provide safety, containment, and emotional availability is compromised, increasing the likelihood of disorganized attachment patterns in children [43,44].

In a more integrative vein, the intergenerational transmission of risk model conceptualizes the effects of maternal trauma as unfolding through interconnected biological, psychological, and relational pathways that shape children’s attachment security and emotion regulation capacities, while also recognizing the buffering role of contextual factors such as social support and reflective functioning [30,45,46].

By contrast, the compensatory model highlights a different adaptive trajectory, suggesting that some mothers respond to traumatic experiences not with withdrawal or dysregulation, but with overprotective or intrusive parenting driven by fear or guilt. Although such behaviors may reflect an attempt to shield the child from further harm, they can inadvertently restrict autonomy and sustain anxiety within the dyad [47,48,49].

Taken together, these models illustrate the multifaceted and sometimes divergent ways in which IPV-related trauma can shape caregiving processes.

Despite substantial evidence linking maternal PTSD and depression to child maladjustment, few studies have examined the specific psychological mechanisms through which these symptoms affect children within a unified explanatory framework. Limited research has simultaneously investigated parenting stress and mentalization or directly compared the relative contributions of PTSD versus depressive symptoms in the same mediation model.

### 1.3. Mediating Mechanisms: Parenting Stress and Polarized Mentalization

Recent research has increasingly focused on parenting stress and mentalization as key mechanisms linking maternal psychopathology to child outcomes [50].

Parenting stress arises when perceived caregiving demands exceed available coping resources. Mothers exposed to IPV often report elevated parenting stress due to ongoing psychological distress, social isolation, and children’s behavioral challenges [51]. High parenting stress is associated with inconsistent discipline, reduced emotional availability, and lower sensitivity, and has been shown to mediate the relationship between maternal PTSD or depression and children’s internalizing and externalizing symptoms [52,53].

Mentalization, or reflective functioning, refers to the ability to understand one’s own and others’ behaviors in terms of underlying mental states [54,55]. Adequate mentalization supports empathy, attunement, and secure attachment. However, trauma may disrupt this capacity. IPV survivors frequently exhibit polarized mentalization, characterized by either excessive certainty or excessive uncertainty about mental states [56]. Excessive certainty involves rigid, overconfident assumptions about others’ intentions, often fostering controlling or punitive parenting. Excessive uncertainty reflects confusion or detachment, leading to emotional withdrawal. Both forms are maladaptive and associated with child adjustment difficulties [57,58].

Contemporary models distinguish between cognitive and affective components of mentalization. PTSD-related hyperarousal may primarily disrupt affective regulation, leading to emotional overload and collapse of reflective functioning. In contrast, depressive symptoms may be more closely linked to cognitive rigidity, negative attributional biases, and reduced epistemic trust. These distinct disruptions may follow partially different pathways in the intergenerational transmission of trauma [49].

Parenting stress and mentalization are best conceptualized as interrelated mechanisms. Maternal trauma and depressive symptoms may be associated with greater caregiving burden, heightening parenting stress. Elevated stress, in turn, undermines reflective functioning by reducing emotional regulation and cognitive flexibility, thereby fostering polarized mentalization.

In this integrated framework, parenting stress represents a proximal contextual amplifier of maternal distress, whereas dysregulated mentalization constitutes the relational pathway through which this distress is transmitted to the child.

### 1.4. The Present Study

Building on the intergenerational transmission of trauma framework, the present study addresses a critical empirical gap by examining parenting stress and polarized mentalization simultaneously within a unified mediation model in IPV-exposed mothers. The study aims to disentangle the distinct and overlapping pathways through which maternal PTSD and depressive symptoms are associated with children’s internalizing and externalizing problems.

**Figure 1:**
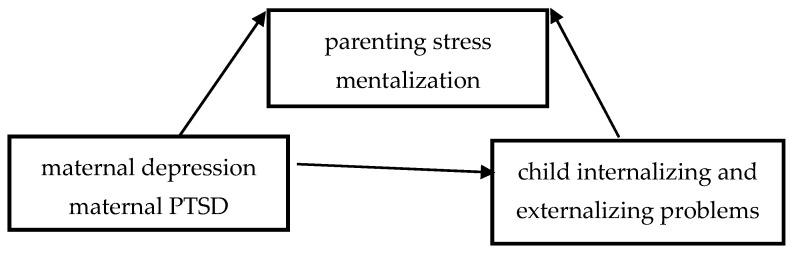
Path diagram of hypothesis conceptual model. Direct and indirect effect of maternal psychopathology on child adjustment through mentalization and parenting stress.

Specifically, we investigated whether parenting stress and polarized mentalization function as interdependent mediators and whether excessive certainty and excessive uncertainty about mental states play differential roles in trauma-related versus depression-related transmission processes. Figure 1 illustrates the conceptual diagrams of this moderated mediation model.

The following hypotheses were tested:

**H1.**  *Higher levels of maternal PTSD and depressive symptoms will be associated with higher levels of children’s internalizing and externalizing problems.*

**H2.**  *Higher levels of maternal PTSD and depressive symptoms will be associated with increased parenting stress and with polarized mentalization profiles (excessive certainty or excessive uncertainty).*

**H3.**  *Parenting stress will mediate the relationship between maternal PTSD and depressive symptoms and children’s internalizing and externalizing problems.*

**H4.**  *Polarized mentalization (both excessive certainty and excessive uncertainty) will mediate the relationship between maternal PTSD and depressive symptoms and children’s internalizing and externalizing problems.*

## 2. Materials and Methods

### 2.1. Participants

Forty-two women who were victims of intimate partner violence (IPV) (M = 43.38 years, SD = 10.56) and their children (N = 42; 52.38% male; means = 8.30 years, SD = 2.53, range = 1–10) participated in the present study. Participants were recruited through Services for Abused Women located in Northern and Central Italy. Inclusion criteria were: (1) having experienced intimate partner violence (IPV); (2) being over 18 years of age; (3) currently living in safe conditions, defined as separation from the abusive partner for at least 30 days; and (4) having at least one preschool- or school-aged child (1–10 years), with only one child per mother included in the analytical sample. Most women were Italian, had a medium-to-high educational level, were employed, and had been married or in a stable cohabiting relationship prior to accessing the service. The majority had experienced chronic and multiple forms of abuse. (see Table 1). Data collection took place between June and September 2025.

### 2.2. Measures

A demographic questionnaire collected information on mothers’ age, educational level, ethnic background, number of children, marital or relationship status, type and duration of intimate partner victimization, as well as children’s age and gender.

Depression symptoms were assessed using the Beck Depression Inventory–II (BDI-II [59]; Italian validation by Ghisi et al. [60]). This 21-item self-report instrument evaluates the cognitive, affective, motivational, and behavioral dimensions of depression. Each item is rated on a 4-point scale ranging from 0 (never) to 3 (always), yielding a total score from 0 to 63. According to the Italian validation, a cut-off score of ≥12 was used to indicate the presence of depression symptoms (minimal depression = 13–19; moderate = 20–28; severe = 29–63). Cronbach’s α coefficients typically range between 0.80 and 0.87 in normative and clinical samples (Beck et al., 1996 [59]); in the present study, internal consistency was α = 0.83.

Post-traumatic stress symptoms were measured using the Los Angeles Symptom Checklist (LASC [61]), a 43-item self-report scale assessing global distress related to trauma exposure, overall PTSD severity, and the severity of specific PTSD symptom clusters (re-experiencing, avoidance/numbing, and hyperarousal). Previous studies reported excellent internal consistency (α = 0.88–0.95 [61]). In the current sample, Cronbach’s α was 0.91.

Parenting stress was evaluated using the Parenting Stress Index–Short Form (PSI–SF [62]; Italian validation by Guarino et al. [63]), a 36-item self-report measure assessing the overall level of stress associated with the parenting role. The PSI–SF provides a Total Stress score and three subscales: Parental Distress (PD), which reflects stress arising from personal factors related to parenting; Parent–Child Dysfunctional Interaction (P-CDI), which captures the perception that the child does not meet parental expectations and that interactions are unrewarding; and Difficult Child (DC), which assesses parental perceptions of the child’s temperament and behavioral characteristics that make parenting challenging. Items are rated on a 5-point Likert scale ranging from 1 (Strongly disagree) to 5 (Strongly agree), with higher scores indicating greater parenting stress. The Total Stress score ranges from 36 to 180. The Italian validation study reported good psychometric properties, with Cronbach’s α values above 0.85 for the total score and satisfactory reliability for the subscales. In this study, the PSI–SF total score was used to assess overall parenting stress, with higher scores reflecting a greater burden associated with the parenting role.

Mentalization was assessed through the Reflective Functioning Questionnaire (RFQ) [56,57]. The RFQ is a self-report and assesses mentalization or reflective function by asking the patient to answer eight items on a Likert scale from 1 (“strongly disagree”) to 7 (“strongly agree”). Scores are then recoded and collapsed into two different subscales: RFQ_certainty (RFQ_c), which reflects an excessive certainty about mental states, and RFQ_uncertainty (RFQ_u), which reflects an excessive uncertainty about self and others’ mental states. The factor structure of the scale has been tested on a sample of patients with eating disorders and borderline PD [56]. In this study, the scale has shown sufficient psychometric properties, with alphas of 0.65 (RFQ_u) and 0.70 (RFQ_c), respectively.

Children’s behavioral and emotional problems were assessed using the Child Behavior Checklist (CBCL/4–18 [64]; Italian version by Frigerio [65]). This 113-item parent-report questionnaire measures behavioral and emotional problems in children and adolescents aged 4–18 years. For younger children, the CBCL/1.5–5 version was used. Each item is rated as 0 (not true), 1 (somewhat or sometimes true), or 2 (very true or often true). The subscales Withdrawn, Somatic Complaints, and Anxious/Depressed form the Internalizing Problems composite, while Delinquent Behavior and Aggressive Behavior form the Externalizing Problems composite. In the present study, internal consistency for the CBCL total score was excellent (Cronbach’s α = 0.92).

### 2.3. Statistical Analyses

Descriptive analyses were conducted to compute mothers’ scores for PTSD, depression, parenting stress, mentalization, and children’s internalizing and externalizing problems. To address the first research hypothesis, correlational analyses were performed among all study variables to examine associations between maternal PTSD and depression symptoms, parenting stress, mentalization, and children’s internalizing and externalizing problems. To test the second and third hypotheses, hierarchical multiple regression analyses were conducted following the procedure outlined by Baron and Kenny [66]. A mediation model was considered valid if four conditions were met: (1) the predictor variable (depression score for the first model; PTSD score for the second) significantly predicted the dependent variable (internalizing problems in the first model; externalizing problems in the second); (2) the predictor variable significantly predicted the mediators (parenting stress and mentalization); (3) the mediators were significantly associated with the dependent variables after controlling for the predictor; and (4) the effect of the predictor on the dependent variable became nonsignificant (full mediation) or was reduced (partial mediation) when the mediator was included in the model. Mediation analyses were conducted using a bootstrapping approach with 1000 resamples and 95% bias-corrected confidence intervals. All statistical analyses were performed using IBM SPSS Statistics version 21.

### 2.4. Procedure

Participants provided written informed consent after receiving a detailed explanation of the study’s aims. They then completed a socio-demographic questionnaire and the research instruments. Participants were informed about potential risks related to the study, including possible distress when responding to items concerning traumatic experiences, and were advised that they could withdraw from participation at any time. Questionnaires were anonymous and completed individually at home. The study adhered to the Ethical Code of Italian Psychologists and was approved by the Ethics Committee of eCampus University (RG 09/2024). All personal data were managed in accordance with the General Data Protection Regulation (GDPR; EU Regulation 2016/679).

## 3. Results

### 3.1. Associations Between Maternal PTSD and Depression Symptoms and Children’s Emotional and Behavioural Problems

Descriptives showed that participants reported a high level of PTSD symptoms (above the LASC cut-off [61]), and they showed a moderate level of depression symptoms (according to the BDI-II cut-off [59]). They also reported elevated parenting stress (M = 120.64, SD = 31.92 [63]) and medium levels of mentalization (certainty: M = 100.61, SD = 18.30; uncertainty: M = 83.92, SD = 26.02). Children exhibited high levels of internalizing and externalizing problems (above cut-off values [67]). Correlational analysis (see Table 2) showed that PTSD and depression symptoms positively correlated with parenting stress and with certainty and uncertainty about one’s own and other’s mental states. Maternal PTSD and depression symptoms were also positively correlated with children’s internalizing and externalizing problems.

### 3.2. Mediation Models for Maternal Depression and PTSD Symptoms Effects on Children’s Emotional and Behavioural Problems

Multiple regression analyses were performed with children’s internalizing and externalizing as dependent variables, depression and PTSD as predictors and parenting stress and polarized mentalization profiles (i.e., excessive certainty or excessive uncertainty regarding one’s own and others’ mental states). The results of the mediation models are presented in Table 3 and Table 4. As standardized regression coefficients (β) are reported and indirect effects were estimated via bootstrapping in separate regression models (ab), the total effect is not expected to equal the sum of the direct and indirect effects. Additivity strictly applies only to unstandardized coefficients estimated within the same model.

#### 3.2.1. Mediation Models for Internalizing Problems

Analysis showed that internalizing problems were first predicted by maternal depression symptoms and secondarily by maternal PTSD. Maternal depression symptoms were found to predict higher levels of children’s internalizing problems. The association was stronger in the presence of higher parenting stress and partially mediated by polarized mentalization profiles, primarily through uncertainty and, secondarily, through excessive certainty. Maternal PTSD symptoms predicted higher levels of children’s internalizing problems, with parenting stress amplifying this association and excessive uncertainty and certainty in mentalization partially mediating the effect (see Table 3).

#### 3.2.2. Mediation Models for Externalizing Problems

Regarding externalizing problems, they are predicted firstly by maternal PTSD and secondarily by depression symptoms. More specifically, maternal depression predicts higher levels of children’s externalizing problems, and its effects are fully mediated by parenting stress, partially mediated by excessive certainty in mentalization and fully mediated by uncertainty about one’s own and others’ mental states. Maternal PTSD symptoms were associated with higher levels of children’s externalizing problems, an effect that was fully mediated by parenting stress and partially mediated by excessive certainty and uncertainty regarding one’s own and others’ mental states (see Table 4).

## 4. Discussion

The present findings corroborate the well-documented association between maternal psychological distress and children’s emotional and behavioral adjustment in intimate partner violence contexts [25,40,67,68]. Maternal psychopathology was associated with children’s emotional and behavioral outcomes both directly and indirectly through heightened parenting stress [69], while maternal mentalization capacities also emerge as a relevant factor within this interplay [49,70,71]. These results align with theoretical models of intergenerational transmission of risk, according to which maternal trauma and depression may compromise caregiving sensitivity and emotional attunement, thereby shaping children’s regulatory and attachment systems [30,45].

Specifically, the finding that maternal depression symptoms were a primary predictor of children’s internalizing problems aligns with a robust body of research establishing the transmission of affective risk from parent to child. Meta-analytic evidence consistently demonstrates that maternal depression is associated with greater internalizing and, to a lesser extent, externalizing difficulties in children across developmental stages [28,72,73]. Depressed mothers may display reduced emotional availability, higher irritability, and impaired responsiveness, which contribute to insecure attachment patterns and the development of anxiety and withdrawal in their offspring [28,74]. That the effect of maternal depression was further amplified by parenting stress is also well supported: parenting stress has repeatedly been shown to mediate or moderate the impact of parental psychopathology on child adjustment [75]. For example, Chen [76] found that parenting stress mediated the association between parental depression and both internalizing and externalizing problems in children, emphasizing the transactional nature of stress within the family system.

The differential pattern observed between maternal depression and PTSD symptoms warrants further theoretical consideration. In the present study, depressive symptoms emerged as the strongest predictor of children’s internalizing problems, whereas maternal PTSD symptoms were associated with both internalizing and externalizing outcomes. This divergence may reflect the distinct affective and behavioral profiles characterizing these conditions. Depression is typically marked by emotional withdrawal, diminished energy, and reduced parental sensitivity, which may foster relational disengagement and increase children’s vulnerability to anxiety, sadness, and social withdrawal. In contrast, PTSD is often characterized by hyperarousal, irritability, emotional outbursts, and heightened vigilance, which may translate into inconsistent discipline, overprotective behaviors, or reactive parenting practices. Such dysregulated interaction patterns can contribute not only to children’s internalizing distress but also to externalizing manifestations, including aggression, impulsivity, and oppositional behaviors, through coercive or emotionally unpredictable parent–child cycles. Thus, while depressive symptomatology may primarily operate through pathways of emotional withdrawal, trauma-related symptoms may activate broader dysregulation pathways that affect multiple domains of child adjustment.

The mediation by polarized mentalization profiles (i.e., excessive uncertainty followed by excessive certainty) is an intriguing and theoretically meaningful extension. Research on parental reflective functioning (mentalization) links both low and dysregulated mentalization to increased risk of children’s emotional and behavioral problems [49,55,77]. This suggests that mothers with depression or trauma-related symptoms may struggle to interpret or regulate their own and their children’s mental states, creating relational misunderstandings and emotional misattunement that heighten child vulnerability to internalizing difficulties [58]. In IPV-exposed contexts, where emotional safety is often compromised, impaired mentalization may further exacerbate the mother’s difficulty in supporting the child’s affect regulation, thereby perpetuating a cycle of distress.

Regarding maternal PTSD symptoms predicting children’s internalizing problems—with parenting stress amplifying the association and excessive uncertainty or excessive certainty partially mediating the effect—these findings are consistent with current models of trauma’s intergenerational transmission [31]. PTSD-related avoidance, hyperarousal, and emotional numbing may be associated with disruptions in parenting functioning, limit emotional communication, and heighten stress in daily caregiving [41,53]. Hartzell et al. [53] specifically found that maternal PTSD severity was linked to increased parenting stress, which in turn predicted higher internalizing and externalizing symptoms in children. This underscores parenting stress as a proximal mechanism through which trauma-related symptoms affect the child’s emotional world.

In sum, our results underscore two interrelated pathways: a direct link from maternal psychopathology (depression or PTSD) to child internalizing problems, and an indirect pathway via parenting stress and compromised mentalization. The evidence supports positioning parenting stress as a key mechanism in both models, and mentalization as a secondary mediator—particularly relevant in the trauma-related pathway (PTSD). These findings converge with a growing body of research emphasizing that interventions targeting parental reflective functioning and stress regulation may buffer the intergenerational transmission of risk and foster resilience in children exposed to IPV [70].

With respect to children’s externalizing problems, the present results indicate that maternal PTSD symptoms represent the strongest predictor, followed by depression symptoms. This finding aligns with previous evidence suggesting that maternal trauma-related distress is particularly associated with children’s externalizing behaviors such as aggression, impulsivity, and rule-breaking [25,41]. PTSD symptoms—especially hyperarousal and emotional dysregulation—may compromise the mother’s ability to respond sensitively to the child’s needs, fostering inconsistent or harsh parenting practices that, in turn, increase the risk of externalizing outcomes in children [40,78]. Moreover, the finding that the effect of maternal PTSD on children’s externalizing problems was fully mediated by parenting stress reinforces the view that chronic stress is a key mediator linking trauma exposure to parenting difficulties [69]. Elevated parenting stress may reduce emotional availability, increase reactivity, and contribute to coercive parent–child cycles that reinforce children’s behavioral dysregulation [79].

Finally, excessive certainty or excessive uncertainty about one’s own and others’ mental states partially mediated this relationship, suggesting that a rigid and overconfident mentalizing style—or, conversely, a lack of epistemic confidence—may hinder mothers’ capacity to flexibly interpret their children’s emotions and intentions [49,77]. Such polarized mentalization could amplify the negative impact of stress and trauma on parenting, thereby increasing behavioral dysregulation in children. This finding underscores the relevance of maternal reflective functioning as both a protective and a risk-modifying factor within the intergenerational transmission of trauma [49,70].

## 5. Conclusions

The present study examined associations between maternal PTSD and depressive symptoms and children’s internalizing and externalizing difficulties in a sample of families exposed to intimate partner violence (IPV). The findings suggest that maternal psychological distress was associated with children’s emotional and behavioral adjustment, both directly and in relation to parenting-related processes such as parenting stress and maternal mentalization. In particular, higher levels of maternal depressive symptoms were associated with children’s internalizing problems, whereas PTSD symptoms were associated with both internalizing and externalizing difficulties. Parenting stress and polarized mentalization were also linked to these associations. These findings highlight the potential relevance of considering parenting stress and maternal mentalization when examining the relationship between maternal psychological distress and children’s adjustment in IPV-exposed families. Although the present results do not support causal interpretations, they suggest that parenting-related processes may represent important correlates in the broader context of maternal trauma and child development.

Several limitations should be acknowledged. The relatively small sample size limits the generalizability of the findings and reduces the statistical power to detect more nuanced patterns of association [80]. In addition, the cross-sectional design precludes causal inferences and does not allow conclusions about the temporal ordering of the observed relationships [28,36]. The exclusive reliance on maternal self-report measures may also have introduced shared method variance and response biases. Furthermore, mentalization was assessed using a global self-report measure, which may not fully capture the complexity of reflective functioning in parent–child interactions [81]. Future research should aim to replicate these findings in larger and more diverse samples, adopt longitudinal designs, and incorporate multi-informant and observational measures of parenting and child functioning. Considering additional contextual factors—such as social support, economic stress, and ongoing exposure to violence—may also help to further clarify the relationships between maternal psychological distress, parenting processes, and child adjustment in families exposed to IPV [7,82].

Overall, the present findings may offer a preliminary contribution to the growing literature examining the associations between maternal mental health, parenting processes, and child adjustment in the context of intimate partner violence. Further research addressing the limitations noted above may help refine current theoretical perspectives and inform future efforts aimed at supporting families exposed to IPV.

## Figures and Tables

**Table 1:** Demographic variables.

Women	42	
**Age (years)**		
mean (SD)	43.38 (10.56)	
min-max	24	58
**Nationality**		
Italian	23	54.8%
Not Italian	19	45.2%
**Education**		
Middle school license	12	28.6%
Degree	21	50%
Post-graduate degree	9	21.4%
**Occupational status**		
employed	31	73.8
unemployed	11	26.2
**Marital status**		
married or cohabiting	29	69.04%
stable partner	13	30.95%
**Children**		42
Gender	M = 22 (52.38%)	F = 20 (47.61%)
**Age (years)**		
mean (SD)	8.30 (2.53)	
min–max	1	10
**Year of victimization**		
<1 year	5	11.90%
>1 year	37	88.10%
Separation from abusing partner		
<6 months	27	65.28%
>6 months	15	35.71%
**Type of victimization**		
Psychological abuse	42	100%
Physical abuse	32	76.19%
Sexual abuse	27	64.28%
More than one type of abuse	42	100%

**Table 2:** Correlational analyses, means, and standard deviations of the investigated variables.

	1	2	3	4	5	6	7	M	SD	RANGE
(1) internalizing	1	0.620 **	0.450 **	0.582 **	0.358 *	0.612 **	0.352 *	10.16	8.26	2–32
(2) externalizing	0.620 **	1	0.362 *	0.303 *	0.298 *	0.314 *	0.411 **	10.92	8.29	4–31
(3) PTSD	0.450 **	0.362 *	1	0.589 **	0.345 *	0.342 *	0.433 **	26.47	11.04	6–47
(4) depression	0.582 **	0.303 *	0.582 **	1	0.348 *	0.457 **	0.381 *	17.69	9.63	1–40
(5) certainty	0.358 *	0.298 *	0.345 *	0.348 *	1	00.002	0.356 *	100.6	18.3	74–130
(6) uncertainty	0.612 **	0.314 *	0.342 *	0.457 **	00.002	1	0.314 *	83.92	26.02	45–168
(7) parenting stress	0.352 *	0.411 **	0.433 **	0.381 *	0.356 *	0.314 *	1	120.6	31.92	52–168

** Sig < 0.01, * Sig < 0.05.

**Table 3:** Indirect effects of maternal PTSD and depression symptoms on children’s internalizing problems through parenting stress, certainty and uncertainty.

**DV Internalizing Problems**				
**PTSD Model**				
**Effect**	**Estimate**	**SE**	**95% Bootstrap CI**	***p***
Total effect (c)	β = 0.38	0.15	—	<0.01
Direct effect (c′)	β = 0.18	0.12	—	<0.05
**Indirect Effects (Bootstrapped ab Estimates):**				
**Mediator**	**Indirect Effect (ab)**	**SE**	**95% Bootstrap CI**	
Parenting stress	0.21	0.14	[0.36, 0.82]	
Certainty	0.18	0.13	[0.28, 0.61]	
Uncertainty	0.20	0.14	[0.26, 0.52]	
**Depression Model**				
**Effect**	**Estimate**	**SE**	**95% Bootstrap CI**	***p***
Total effect (c)	β = 0.50	0.17	—	<0.01
Direct effect (c′)	β = 0.32	0.15	—	<0.05
**Indirect Effects (Bootstrapped ab Estimates):**				
**Mediator**	**Indirect Effect (ab)**	**SE**	**95% Bootstrap CI**	
Parenting stress	0.34	0.13	[0.24, 0.72]	
Certainty	0.25	0.13	[0.26, 0.69]	
Uncertainty	0.37	0.15	[0.20, 0.64]	
(Model R^2^ = 0.35, *p* < 0.01)				

**Table 4:** Indirect effects of maternal PTSD and depression symptoms on children’s externalizing problems through parenting stress, certainty and uncertainty.

**DV: Externalizing Problems**				
**PTSD Model**				
**Effect**	**Estimate**	**SE**	**95% Bootstrap CI**	***p***
Total effect (c)	β = 0.36	0.15	—	<0.01
Direct effect (c′)	β = 0.20	0.12	—	<0.05
**Indirect Effects (Bootstrapped ab Estimates):**				
**Mediator**	**Indirect Effect (ab)**	**SE**	**95% Bootstrap CI**	
Parenting stress	0.31	0.13	[0.28, 0.65]	
Certainty	0.32	0.13	[0.31, 0.68]	
Uncertainty	0.26	0.12	[0.26, 0.71]	
(Model R^2^ = 0.21, *p* < 0.01)				
**Depression Model**				
**Effect**	**Estimate**	**SE**	**95% Bootstrap CI**	***p***
Total effect (c)	β = 0.30	0.12	—	<0.05
Direct effect (c′)	β = 0.26	0.12	—	<0.05
**Indirect Effects (Bootstrapped ab Estimates):**				
**Mediator**	**Indirect Effect (ab)**	**SE**	**95% Bootstrap CI**	
Parenting stress	0.29	0.13	[0.22, 0.75]	
Certainty	0.28	0.12	[0.26, 0.69]	
Uncertainty	0.27	0.12	[0.21, 0.63]	
(Model R^2^ = 0.20, *p* < 0.05)				

## Data Availability

The data presented in this study are available upon reasonable request from the corresponding author. This is due to the presence of sensitive participant information, which our university’s Ethics Committee advises not to store in online repositories.
